# Therapeutic Efficacy and Safety of Safflower Injection in the Treatment of Acute Coronary Syndrome

**DOI:** 10.1155/2021/6617772

**Published:** 2021-03-16

**Authors:** Qiang Lu, Jiamin Xu, Qian Li, Wenzhen Wu, Yuling Wu, Jianhui Xie, Xiaobo Yang

**Affiliations:** ^1^Department of Pharmaceutical Sciences, Zunyi Medical University, Zhuhai Campus, Zhuhai 519041, China; ^2^The Second School of Medicine, Guangzhou University of Chinese Medicine, Guangzhou 510120, China; ^3^The Second Affiliated Hospital of Guangzhou University of Chinese Medicine, Guangzhou 510120, China; ^4^State Key Laboratory of Dampness Syndrome of Chinese Medicine, The Second Affiliated Hospital of Guangzhou University of Chinese Medicine, Guangzhou 510120, China; ^5^Guangdong Provincial Key Laboratory of Clinical Research on Traditional Chinese Medicine Syndrome, Guangzhou 510120, China

## Abstract

**Background:**

Safflower injection (SFI), a popular Chinese patent drug, is commonly used to treat acute coronary syndromes (ACSs) in China. The research seeks to scientifically estimate the clinical efficacy of SFI for ACS patients.

**Methods:**

Eight electronic databases were retrieved for eligible research from the founding date to September 8, 2020. Odds ratio (OR) was adopted to assess the total effective rate, ECG improvement, and adverse reaction, and mean difference (MD) was used for assessing the hemorheology indexes as well as the LVEF.

**Results:**

Sixteen randomized controlled trials involving 1620 sufferers with ACS were incorporated. The outcomes showed that, in comparison to conventional medication alone, SFI combined with conventional treatment remarkably enhanced the total effective rate (OR = 3.66, 95% CI [2.73, 4.90], *P* < 0.00001), ECG improvement (OR = 2.85, 95% CI [2.04, 3.99], *P* < 0.00001), and LVEF (MD = 5.13, 95% CI [3.73, 6.53], *P* < 0.00001). Moreover, SFI combined with conventional treatment significantly decreased hemorheology indexes including BV (MD = −0.95, 95% CI [−1.76, −0.13], *P*=0.02), HCT (MD = −2.37, 95% CI [−3.25, −1.50], *P* < 0.00001), FIB (MD = −0.44, 95% CI [−0.60, −0.29], *P* < 0.00001), and PAR (OR = −7.65, 95% CI [−10.16, −5.14], *P* < 0.00001). However, no notable contrast was observed to link the experimental and the control team for PV (MD = −0.42, 95% CI [−0.83, 0.00], *P*=0.05) and adverse reactions (OR = 0.59, 95% CI [0.13, 2.74], *P*=0.50).

**Conclusion:**

Despite the limitations that existed in this meta-analysis, the outcomes demonstrated that SFI and conventional combined medication is an effective and relatively safe therapy for ACS sufferers.

## 1. Introduction

Acute coronary syndrome (ACS) is a category of clinical syndromes resulting from acute myocardial ischemia. These syndromes include ST-segment elevation myocardial infarction, non-ST-segment elevation myocardial infarction, and unstable angina pectoris [1]. The main pathological basis of ACS is the formation of complete or incomplete occlusive thrombosis induced by the breakage or erosion of unstable atherosclerotic plaques in coronary arteries [2]. ACS is mainly characterized by acute onset, rapid progression, and high mortality [3, 4]. Presently, cardiovascular disease (CVD) is attributed to almost a third of global fatalities, and the most serious of them all is ACS, which results in five million hospitalizations and two hundred and seventy billion dollars cost each year in Europe and America [5, 6].

The treatment of ACS mainly includes medication, surgery, and intervention operation [7]. Among them, medication is the popular procedure [8]. Western medicines for the conventional treatment of ACS include statins, *β*-blockers, nitrates, calcium channel restrictors, and angiotensin-converting enzyme inhibitors [9]. However, the clinical efficacy of the conventional western treatments is still limited, and the adverse reactions resulted from these cannot be overlooked. For example, side effects such as gastrointestinal reactions, headaches, and hypotension are prone to occur during the treatment of ACS using nitrates [10]. Therefore, how to improve the efficacy of conventional medication on ACS and relieve the adverse reactions is a direction that clinical staff should strive for.

Safflower injection (SFI) is a popular Chinese patent drug that has been authorized by the China Food and Drug Administration. It is prepared from *Carthami flos* by water extraction and alcohol precipitation [11]. The major components of SFI are carthamin yellow, which belongs to chalcones. According to Traditional Chinese Medicine (TCM) theory, the manifestation of ACS is associated with stagnant blood block [12]. However, SFI is good at promoting blood circulation by removing blood stasis, which is contributed to relieve stagnant blood block. Modern pharmacological studies also have indicated that SFI could expand the coronary artery, protect the myocardium, and eliminate free radicals which reduce the appearance of angina [13]. SFI has been linked to absolute efficacy on sufferers with coronary heart disease, hypertension, cerebral infarction, and other CVDs [14].

Along with the development of integration of traditional Chinese and Western medicine, SFI combined with conventional treatment (Western medicines) was increasingly prescribed for treating ACS over the past decades, and some studies showed that it might bring beneficial effects to the patients [15]. However, a higher percentage of the clinical studies have not given enough proof from the small sample sizes. Systematic evidence which could prove the efficacy and safety is demanded extremely. Thus, the meta-analysis was performed by roundly assessing the efficacy of SFI and conventional combined treatment for ACS compared to single conventional treatment, with the hope of providing a statistical record of this combined medication.

## 2. Methodology

### 2.1. Search Strategy

The PRISMA statement was used to form a basis for the meta-analysis [16]. Randomized controlled trials (RCTs) were independently searched and retrieved by two investigators (Qiang Lu and Jiamin Xu). The including databases were used from the formation date to September 8, 2020: PubMed, Embase, the Cochrane Library, Web of Science (WOS), China National Knowledge Infrastructure (CNKI), China Biology Medicine disc (CBMdisc), Wanfang Data, and VIP medicine information system (VMIS). Two different strategies were used in the literature search. For the English databases, the following retrieval terms were used in combination: (“Safflower injection” OR “Honghua injection”) AND (“unstable angina” OR “acute myocardial infarction” OR “acute coronary syndrome”). The following keywords were searched in combined ways for Chinese databases: [“hong hua zhu she ye (in Chinese)”] AND [“bu wen ding xin jiao tong (in Chinese)” OR “ji xing xin ji geng si (in Chinese)” OR “ji xing guan mai zong he zheng (in Chinese)”]. Research studies published using either English or Chinese were taken into account.

### 2.2. Inclusion Criteria

After consulting with several cardiologists, the inclusion criteria were formulated as follows: subjects were confirmed to suffer from ACS according to the cardiovascular disease examination method formulated by the Chinese Medical Society (CMA) as well as the American Heart Association (AHA) with randomized controlled trials (RCTs) [17, 18]; all studies enumerated were detailed as RCTs; SFI administered as the single Chinese patent medicine in RCTs; sufferers in the experimental team had administration of combined therapy of SFI and conventional treatment, while those in the control team got the conventional treatment alone; outcomes of each study had not less than one of these indices: total effective rate, electrocardiogram (ECG) improvement, hemorheology indexes including blood viscosity (BV), hematocrit (HCT), fibrinogen (FIB), plasma viscosity (PV), and platelet aggregation rate (PAR), left ventricular ejection fraction (LVEF), and adverse reactions.

### 2.3. Exclusion Criteria

This was formulated as follows: reviews, case report, animal experiments, editorials, and unrelated clinical studies; research studies were found not to be RCTs or diagnosis standards were unclear; studies containing patients diagnosed with stable angina; the interference of ACS sufferers was not accordant; and for the studies with information replication, the subsequent publications were considered as data fraud and were then denied once the authors were unreachable.

### 2.4. Data Extraction and Quality Assessment

Details on relevant studies which include author names, issuance year, sample capacity, intervening measures, and outcomes were generalized. In accordance to the Cochrane Handbook for Systematic Reviews of Interventions, quality evaluation of the incorporated research studies was separately carried out by the researchers (Qiang Lu and Qian Li) using the risk of bias table from Review Manager 5.3 [19]. From this information, seven types of biases were derived. Each of them was evaluated using three levels: low risk of bias, unclear, and high risk of bias. “Low risk of bias” shows the illustration of procedures was sufficient or accurate, whereas “high risk of bias” indicates insufficient or inaccurate. When insufficient detail was presented in the research and we could not decide whether it is “high risk” or “low risk,” the object was described as “unclear.” Data extraction and study evaluation inconsistencies were judged through requited analysis or discourse with a third party (Xiaobo Yang).

### 2.5. Statistical Analysis

Review Manager 5.3 (Cochrane Collaboration) was employed to process the extracted data from the relevant studies [19]. Resulting measures which include total effective rate, ECG improvement, and adverse reactions were referred to as dichotomous variables. These were accorded to be the odds ratio (OR) having a 95% confidence interval (CI). The hemorheology indexes (BV, PV, HCT, FIB, and PAR) and LVEF were continuous variables that were given as the mean difference (MD) with 95% CI. Chi-square analysis was used to examine the heterogeneity among studies, and the *I*^2^ statistic was employed to evaluate the level of heterogeneity. A fixed-effect model was employed to process data with low heterogeneity (*P* > 0.1 and *I*^2^ < 50%), and data having high heterogeneity (*P* < 0.1 or *I*^2^ > 50%) were assessed by a random-effect model [20]. The risk of publication bias was illustrated in the selected studies using a funnel plot.

## 3. Results

### 3.1. Study Selection

One hundred and fifty-three possible data from Chinese databases were selected in the initial analysis, and similar data were not recovered from English databases. Eighty-five replicated articles were removed as a result of overlapping of the database scope. A sum of 68 studies was acquired for title or abstract check up, and 27 research studies were eliminated due to irrelevant subjects. Forty-one articles were put aside to examine complete information.

**Figure 1:**
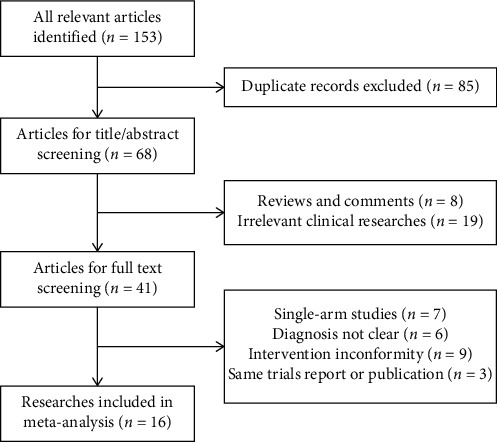
Flow chart of trial searching and screening for this meta-analysis.

In the inspection of complete information, 25 pieces of research were excluded as a result of the following: 7 studies were single-arm designs, diagnosis in 6 researches was unclear, 9 studies brought up unsuitable interferences, and 3 trials severally presented similar records with another publication. In the end, there were sixteen pieces of study used for this meta-analysis (Figure 1).

### 3.2. Study Features

Due to SFI-based treatments being mainly used in Chinese Medicine or Integrative Medicine, 16 relevant studies consisting of 1620 sufferers were documented in Chinese databases from 2003 to 2020. The experimental team had 832 patients, while the control team had 788 patients. The total number of males (59.1%) is higher than that of females in the included studies, and the mean age of all the patients was approximately 60.9, ranging from 37 to 83. All the adopted trials were RCTs with a contrast between the SFI and conventional combined treatment and single conventional treatment, and there were some similarities and differences in the conventional treatments. The dosage of SFI was between 15 to 40 mL/day by intravenous drip, and most researchers documented the span of drug administration to be 2 weeks. An absolute dissimilarity was not seen evident between the two groups from fundamental data (Table 1).

### 3.3. Quality Assessment of the Eligible Studies

**Figure 2:**
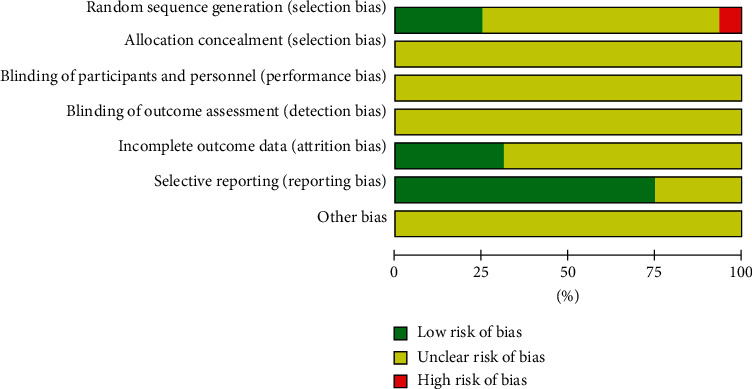
Methodological quality evaluation for the risk of bias in the eligible researches.

The methodological quality of the selected research studies was judged by the Cochrane risk of bias assessment and presented universally low. All the included studies were parallelly designed [21–36]. Eleven of the 16 trials identified the allocation sequence generation in the absence of indicating the concrete procedure [21, 22, 25–30, 33–35]. Only 4 research studies [23, 24, 32,36] showed that they were randomly grouped in accordance to the random number table procedure. All the selected studies did not explain allocation secrecy, blinding of sufferers, and result evaluation. Five trials [23, 25, 27, 32, 36] were at a low risk of attrition bias for giving whole outcome information. Twelve pieces of study [21, 23–26, 28–32, 35, 36] documenting the result of comprehensive indexes indicated a low risk of reporting bias. The risk of bias graph is described in Figure 2.

### 3.4. Total Effective Rate

**Figure 3:**
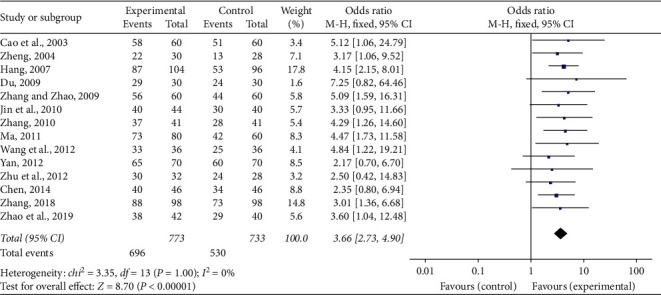
Forest plot of total effective rate of SFI plus conventional treatment compared to single conventional treatment for ACS. *I*2 and *P* are the criteria for the heterogeneity test, ◆: pooled odds ratio, –■–: odds ratio, and 95% CI.

Fourteen of 16 research studies [21–23, 25–33, 35, 36] compared the total effective rate between SFI together with conventional medication and conventional medication alone. Meta-analysis of the 14 researches employing a fixed-effect model showed that the combined administration of SFI and conventional medication notably enhanced the total effective rate than single conventional medication in treating ACS (OR = 3.66, 95% CI [2.73, 4.90], *P* < 0.00001). No statistically notable heterogeneity (*P*=1, *I*^2^ = 0%) was detected among studies individually (Figure 3).

### 3.5. ECG Improvement

**Figure 4:**
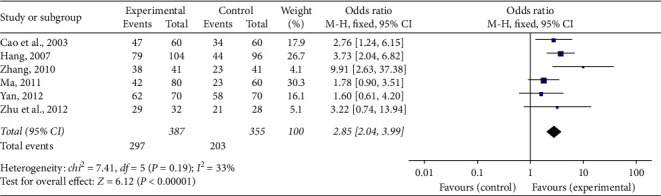
Forest plot of ECG improvement of SFI plus conventional treatment compared to conventional treatment alone for ACS. *I*2 and *P* are the criteria for the heterogeneity test, ◆: pooled odds ratio, –■–: odds ratio, and 95% CI.

Six of the included research studies [21, 23, 28, 29, 31, 32] reported ECG improvement. Meta-analysis using a fixed-effect model indicated that the number of participants with ECG improvement increased remarkably in the experimental team contrasted to the control team (OR = 2.85, 95% CI [2.04, 3.99], *P* < 0.00001). There was no statistically notable heterogeneity (*P*=0.19, *I*^2^ = 33%) in the meta-analysis (Figure 4).

### 3.6. Hemorheology Indexes

**Figure 5:**
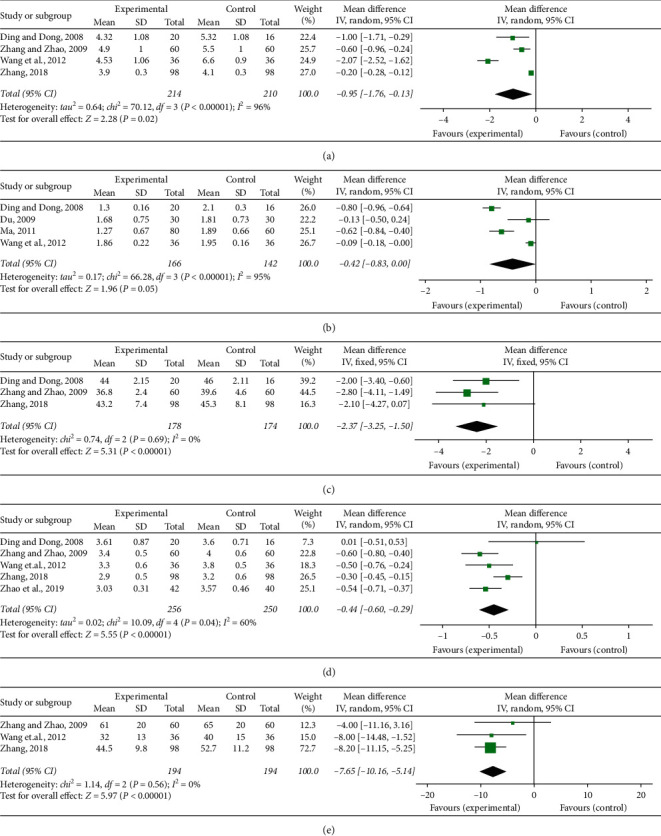
Forest plot of comparison in two groups for hemorheology indexes. (a) Blood viscosity; (b) plasma viscosity; (c) hematocrit; (d) fibrinogen level; and (e) platelet aggregation rate. *I*2 and *P* are the criteria for the heterogeneity test, ◆: pooled mean difference, –■–: mean difference, and 95% CI.

BV, PV, HCT, FIB, and PAR were regarded as indexes of blood rheology recorded in the relevant studies. Four of the tests [24, 26, 30, 35] mentioned the detection of BV. Remarkable heterogeneity was discovered among the studies (*P* < 0.00001, *I*^2^ = 96%); therefore, a random-effect model was employed to perform a meta-analysis which indicated that SFI and conventional combined administration notably lessened BV (MD = −0.95, 95% CI [−1.76, −0.13], *P*=0.02) (Figure 5(a)).

Four studies [24, 25, 29, 30] provided the values of PV. There existed dramatically notable heterogeneity (*P* < 0.00001, *I*^2^ = 95%) among individual studies, and a meta-analysis employing a random-effect model manifested no difference linking the PV in the experimental and control teams (MD = −0.42, 95% CI [−0.83, 0.00], *P*=0.05) (Figure 5(b)).

Three research studies [24, 26, 35] reported the detection of HCT. A fixed-effect meta-analysis indicated SFI together with conventional medication greatly decreased HCT compared to single conventional medication (MD = −2.37, 95% CI [−3.25, −1.50], *P* < 0.00001). There was no notable heterogeneity detected in the research studies (*P*=0.69, *I*^2^ = 0%) (Figure 5(c)).

Five studies [24, 26, 30, 35, 36] indicated FIB concentration in blood plasma. There was a statistically prominent heterogeneity discovered from the trials (*P*=0.04, *I*^2^ = 60%). A random-effect meta-analysis was undertaken to indicate that the combination of SFI and conventional medication notably lowered FIB concentration in blood plasma (MD = −0.44, 95% CI [−0.60, −0.29], *P* < 0.00001) (Figure 5(d)).

Three trials [26, 30, 35] recorded the detection of PAR in blood. No heterogeneity was found (*P*=0.56, *I*^2^ = 0%) among the researches, so a fixed-effect model meta-analysis was conducted. The pooled OR indicated the SFI-conventional combined medication notably decreased PAR in contrast to the conventional medication alone (OR = −7.65, 95% CI [−10.16, −5.14], *P* < 0.00001) (Figure 5(e)).

### 3.7. LVEF

**Figure 6:**
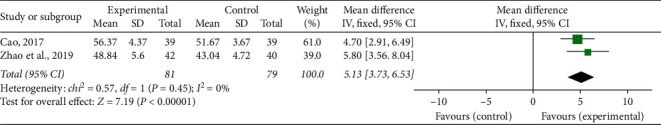
Forest plot of comparison in two groups for LVEF. *I*2 and *P* are the criteria for the heterogeneity test, ◆: pooled mean difference, –■–: mean difference, and 95% CI.

Two studies [34, 36] mentioned the investigation on LVEF. There was no heterogeneity checked in the meta-analysis, and so, a fixed-effect model was adopted (*P*=0.45, *I*^2^ = 0%). An OR with 95% CI was used to show the contrast link of LVEF in the experimental and control teams (MD = 5.13, 95% CI [3.73, 6.53], *P* < 0.00001). It indicated that SFI could notably extend LVEF for ACS patients (Figure 6).

### 3.8. Adverse Reactions

**Figure 7:**
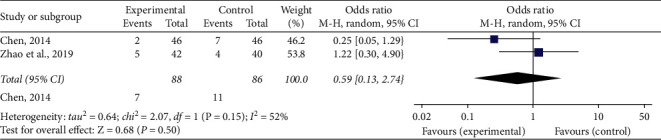
Forest plot of comparison in two groups for the incidence of adverse reactions. *I*2 and *P* are the criteria for the heterogeneity test, ◆: pooled odds ratio, –■–: odds ratio, and 95% CI.

One [29] of the included researches indicated no clear adverse reaction took place after administration of medication, and two [33, 36] indicated occurrences of adverse reactions. These reactions were characterized by nausea and vomiting, flushing, headache, and diarrhea. A significant heterogeneity (*P*=0.15, *I*^2^ = 52%) was detected in the researches, so a random-effect model was employed to carry out this meta-analysis. The merged OR with 95% CI indicated no difference in the occurrence of adverse reactions linked the experimental and control teams (OR = 0.59, 95% CI [0.13, 2.74], *P*=0.50) (Figure 7).

### 3.9. Publication Bias

**Figure 8:**
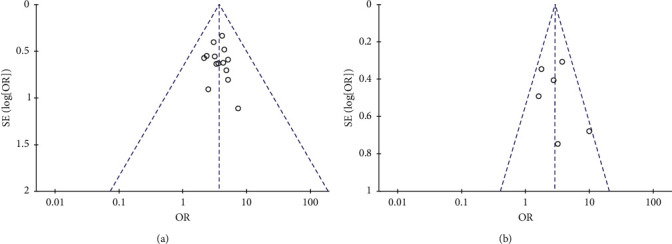
Funnel plot for the publication bias of (a) total effective rate and (b) ECG improvement.

A funnel plot was used to estimate the publication bias. There were 14 and 6 pieces of research severally brought into the funnel plots of total effective rate and ECG improvement. As indicated in Figure 8, both of the plots were symmetrical, showing no publication bias in the included trials.

## 4. Discussion

Cardiovascular disease (CVD) is caused by multiple risk factors such as development standards of life, lifestyle switches, aging of the population, and the gradually changing environment [37]. The morbidity and mortality of such disease has been consistently high, and the burden of prevention and treatment of CVD is increasing. It has become a central public health topic. ACS is the toughest form of CVD with a high handicap rate, mortality rate, and other health conditions which is a serious threat to human health [38, 39]. The main treatment methods of ACS include life intervention, drug treatment, percutaneous coronary intervention, and comprehensive treatment [40, 41]. Percutaneous coronary intervention works well, but it is expensive and exceeds the affordability of many patients. Therefore, relatively inexpensive and effective drug treatment is still a practical solution. However, the therapeutic effects of Western medicines are limited, and some adverse reactions appeared during the period of the treatment. Therefore, more efficacious and safe treatments are quickly needed for these patients in China and throughout the globe.

Over the years, Chinese medics have been seeking for more effective treatments for ACS. TCM has been used to treat coronary heart disease (including ACS) for more than 2000 years. The curative efficacy of Chinese medicines in dealing with ACS is clear and more potent than some Western drugs, and Chinese medicines have less toxicity and fewer side effects. Thus, use of the Chinese medicines on ACS is also as important. Together with the improvement of contemporary pharmacy techniques, all kinds of medicinal preparations for treating patients of ACS that based on classical prescriptions of Chinese medicines have been greatly developed [42]. *Carthami flos* (namely, the dry flowers of *Carthamus tinctorius* L.) is a Chinese traditional medicine, which has been applied in the clinic since the Han Dynasty. Its main function is activating blood to promote menstruation and eliminating stasis to stop the pain [43]. SFI was successfully developed from *Carthami flos* in 1973 and has been widely used for more than 40 years [44]. It has been consistently demonstrated that SFI can effectively treat many CVD [45]. However, there is no extensive and systematic assessment of SFI for the remedy of ACS in accordance with general international standards. Thus, this research intends to give a globally recognized system evaluation of the clinical effect of SFI for the treatment of ACS.

The pathogenesis of ACS mainly involves the atherosclerotic plaque rupture, platelet aggregation, and thrombosis, in which plaque rupture is the dominant initiating event [40]. It was reported that BV could be increased due to elevated hemorheological parameters, such as HCT and FIB. Increased BV may result in high rapture forces at the vascular endothelium and promote the breakage of occlusive plaque [46]. Lee et al. also found that elevated BV in ACS patients was related to coronary plaque rupture; thus, BV may be a therapeutic target for the treatment of ACS [47]. Besides, studies had shown that a pathological platelet aggregation is a critical event promoting intravascular thrombus formation, and the inhibition of platelet aggregation has been a drug development target in ACS [48].

This meta-analysis is the first research conducted to assess the safety and efficacy of SFI for curing ACS systematically. Total effective rate and ECG improvement were used to evaluate the efficacy of SFI for ACS. In comparison to single conventional therapy, SFI-conventional combination therapy was linked with a notably higher total effective rate and ECG improvement (*P* < 0.00001). Hemorheology indexes, such as BV, PV, HCT, FIB, and PAR, were employed to investigate the flow and deformation of blood in ACS patients. In comparison to single conventional therapy, SFI-conventional combination therapy was linked to notably lower BV, HCT, FIB, and PAR (*P* < 0.05). This showed that SFI improved the antithrombotic and anticoagulation actions. LVEF was employed to evaluate the cardiac function of ACS sufferers. Compared to single conventional therapy, SFI-conventional combination therapy was linked with a notably higher LVEF (*P* < 0.00001). There was, however, no contrast in the adverse reactions linking the experimental and control teams (*P*=0.5). Because SFI did not reduce the incidence of adverse reactions caused by Western medicines, a temporary conclusion could be reached only that SFI is almost safe.

Extensive searching and stern procedures were applied to select trials and look into the medical efficacy and safety linked with SFI administration. However, many possible restrictions were present in the meta-analysis and need to be contemplated. Firstly, despite adoption of a comprehensive searching strategy to lessen the publication bias as far as possible, there was still a certain level of selective bias which this meta-analysis only narrowed down to Chinese and English databases, and no mention of studies written in different languages was made. Secondly, all relevant studies were conducted in China, and the majority of the patients were Chinese. However, population diversity is important when doing such a study for one to get better convincing and well-grounded results. Thirdly, the majority of the relevant studies exhibited relatively low methodological quality. Eleven of the 16 researches employed “randomization,” whereas no mention of the particular procedure used was made. Furthermore, all the relevant studies did not indicate allocation concealment and blindness. Fourthly, we did not get more details of the studies from the authors through telephone and electronic mail. Fifthly, there was a statistically notable heterogeneity found in the indexes of hemorheology including BV, PV, and FIB. It is rather hard to investigate the heterogeneity in the results of continuous variables. We cannot conduct a subgroup analyses for the few studies giving hemorheology indexes and also did not find the origins of the heterogeneity after carrying out sensitivity analyses. It can be inferred that the heterogeneity resulted from two or more factors, which include sex, age, and period of therapy. Finally, treatment safety is important to come up with other treatments. However, there were only two of the 16 studies which informed adverse reactions. In light of the restraints that existed in the meta-analysis, high-quality and large-scale RCTs, with a fine design and methodology, are required to study the efficacy and safety of SFI for ACS in time to come.

## 5. Conclusions

The therapy of ACS has become a global challenge. The combination of Chinese patent drug SFI and conventional medication may bring advantageous effects to improve blood rheology and cardiac function of sufferers with ACS. Thus, it is recommended to consider SFI in the conventional treatment of ACS. It is worthy of paying attention to the limitations in the meta-analysis. Furthermore, the therapeutic effect and safety of SFI as an adjunctive therapy for ACS still requires methodologically strict studies to prove.

## Figures and Tables

**Table 1:** Characteristics of the included studies.

Study ID	Number (E/C)	Gender (E/C)	Intervention	Control (conventional treatment)	SFI dosage	Duration	Outcome measures
Cao et al. [21]	60/60	67/53	SFI + control	Aspirin and nitroglycerin	20 mL, q.d.	14 days	TER and ECG improvement
Zheng [22]	30/28	38/20	SFI + control	Aspirin, nitroglycerin, metoprolol, ACEI, isosorbide dinitrate, etc.	20 mL, q.d.	10 days	TER
Hang [23]	104/96	123/77	SFI + control	Aspirin, nitrates, ACEI, statins, metoprolol, etc.	20 mL, q.d.	10 days	TER and ECG improvement
Ding and Dong [24]	20/16	22/14	SFI + control	Aspirin, nitrates, ACEI, etc.	20 mL, q.d.	14 days	HR indexes
Du [25]	30/30	34/26	SFI + control	Aspirin, nitrates, *β*-blockers, calcium antagonists, etc.	20 mL, b.i.d.	10 days	TER and HR indexes
Zhang and Zhao [26]	60/60	80/40	SFI + control	Aspirin, isosorbide dinitrate, metoprolol, etc.	20 mL, q.d.	14 days	TER and HR indexes
Jin et al. [27]	44/40	51/33	SFI + control	Aspirin, isosorbide mononitrate, statins, low molecular heparin, etc.	40 mL, q.d.	14 days	TER
Zhang [28]	41/41	54/28	SFI + control	Aspirin, nitrates, *β*-blockers, calcium antagonists, etc.	20 mL, q.d.	15 days	TER and ECG improvement
Ma [29]	80/60	73/67	SFI + control	Aspirin and nitrates	20 mL, q.d.	15 days	TER, HR indexes, and ECG improvement
Wang et al. [30]	36/36	43/29	SFI + control	Aspirin, nitrates, calcium antagonists, etc.	20 mL, q.d.	14 days	TER and HR indexes
Yan [31]	70/70	82/58	SFI + control	Aspirin, isosorbide mononitrate, simvastatin, etc.	30 mL, q.d.	14 days	TER and ECG improvement
Zhu et al. [32]	32/28	45/15	SFI + control	Aspirin, nitrates, clopidogrel, statins, low molecular heparin, etc.	40 mL, q.d.	14 days	TER and ECG improvement
Chen [33]	46/46	50/42	SFI + control	Aspirin, atorvastatin, etc.	20 mL, q.d.	14 days	TER and ARs
Cao [34]	39/39	47/31	SFI + control	Aspirin, atorvastatin, *β*-blockers, etc.	20 mL, q.d.	15 days	LVEF
Zhang [35]	98/98	103/93	SFI + control	Aspirin, isosorbide mononitrate, ACEI, calcium antagonists, etc.	20 mL, q.d.	14 days	TER and HR indexes
Zhao et al. [36]	42/40	45/37	SFI + control	Aspirin, simvastatin, clopidogrel, etc.	15 mL, q.d.	28 days	TER, HR indexes, LVEF, and ARs

E, experimental group; C, control group; SFI, safflower injection; ACEI, angiotensin-converting enzyme inhibitors; q.d., once a day; b.i.d., twice a day; TER, total effective rate; ECG, electrocardiogram; HR, hemorheology; LVEF, left ventricular ejection fraction; AR, adverse reactions.

## Data Availability

The data used to support the findings of this study are included within the article.

## Abbreviations

ACS: Acute coronary syndrome
BV: Blood viscosity
CVD: Cardiovascular disease
ECG: Electrocardiogram
FIB: Fibrinogen
HCT: Hematocrit
LVEF: Left ventricular ejection fraction
MD: Mean difference
OR: Odds ratio
PAR: Platelet aggregation rate
PV: Plasma viscosity
RCTs: Randomized controlled trials
SFI: Safflower injection
TCM: Traditional Chinese Medicine.
